# Family caregivers’ concerns about advance care planning for home-dwelling people with dementia: a cross-sectional observational study in Japan

**DOI:** 10.1186/s12904-022-01008-0

**Published:** 2022-06-27

**Authors:** Miharu Nakanishi, Taeko Nakashima, Yuki Miyamoto, Syudo Yamasaki, Atsushi Nishida

**Affiliations:** 1 Department of Psychiatric Nursing, Tohoku University Graduate School of Medicine, Miyagi, Japan; 2 Research Center for Social Science & Medicine, Tokyo Metropolitan Institute of Medical Science, Tokyo, Japan; 3 Department of Social Healthcare and Business, Nihon Fukushi University, Aichi, Japan; 4 Department of Psychiatric Nursing, Graduate School of Medicine, The University of Tokyo, Tokyo, Japan

**Keywords:** Advance care planning, Attitudes, Dementia, Family caregiver, Psychological well-being, Palliative care

## Abstract

**Background:**

The importance of advance care planning for people with dementia has increased during the Coronavirus Disease 2019 Pandemic. However, family caregivers may have concerns about having conversations regarding advance care planning with their loved ones, which may hinder the initiation of such planning. This study investigated family caregivers’ concerns regarding conducting advance care planning for home-dwelling individuals with dementia.

**Methods:**

A prospective cross-sectional study compared the level of family-caregiver concern between those who had initiated advance care planning and those who did not. In June 2021, an internet-based questionnaire survey was administered to Japan-based family caregivers of persons with dementia. Registered members of a Japan-based survey company were recruited; inclusion criteria were being aged 40 years or older and having been a primary, non-professional caregiver of a family member with dementia. Respondents rated their level of agreement with six statements regarding advance-care-planning-related concerns. Respondents also reported their psychological well-being using the WHO-5 Well-Being Index.

**Results:**

Overall, 379 family caregivers participated in this survey. Of these, 155 (40.9%) reported that their loved ones had initiated advance care planning, of whom 88 (56.8%) stated that care professionals were involved in the advance-care-planning conversations. The level of family-caregiver concern was significantly lower when the loved one initiated the conversation concerning advance care planning. After adjusting for the characteristics of persons with dementia and their caregivers, family caregivers with lower psychological well-being showed significantly higher levels of concern.

**Conclusions:**

Family caregivers reported concerns regarding conducting advance care planning. There is a need for educational and clinical strategies that encourage professionals to address the psychological needs of family caregivers.

## Background

The Coronavirus Disease 2019 (COVID-19) Pandemic has created health-care crises around the world and caused the closure of social support services, resulting in reductions in access to health care and limitations on resources; for family caregivers of persons with dementia, these impacts have created ambiguity and anxiety, as well as burden and stress [1–3]. Prior to the COVID-19 Pandemic, people with dementia showed a higher risk of physical and cognitive decline following hospital admission than people without dementia [4]; this situation has worsened with the arrival of COVID-19, with patients with dementia who are hospitalised with COVID-19 showing lower treatment intensity and higher mortality when compared to other patients hospitalised with COVID-19 [5]. This indicates that, when people with dementia experience issues that could necessitate hospitalisation, the people with dementia and their family caregivers will need to make quick decisions under highly stressful circumstances regarding whether to go to hospital or remain at home. The acute risk of cognitive deterioration and mortality among people with dementia has created an urgent need for advance care planning (ACP) [6, 7]. It is generally recommended that conversations regarding ACP be initiated when the person with dementia has sufficient mental capacity to consider his/her preferences and make decisions regarding his/her future [4, 8]. ACP conversations usually involve family caregivers, as these caregivers are often required to act as proxy decision-makers when, in the future, the loved one lacks the capacity to do so [9].

Family caregivers may have concerns about having conversations regarding ACP with persons with dementia, and this can create a barrier to the initiation of ACP. Although people with dementia appear to show little distress about ACP conversations, family caregivers often find the prospect of raising this topic stressful and challenging [10]. In particular, some family caregivers are concerned that ACP conversations will cause stress and anxiety for the persons with dementia [11]. However, existing findings regarding family caregivers’ perceptions of ACP-related conversations with people with dementia have generally been based on qualitative data [12]. The existing qualitative data used a relatively small sample size from five to 46 family caregivers [11] mainly located in the United States, United Kingdom, and other European countries [13], or countries other than Japan [11–13], and these studies were conducted before the COVID-19 pandemic [10–13]. There is little quantitative evidence concerning such caregivers’ levels of concerns regarding conducting ACP with their loved ones. The quantified level of family caregivers’ concerns can be used for outcome measures to inform the effectiveness of psychosocial interventions to promote ACP initiation in the future. Such data are critical for promoting ACP for people with dementia during the COVID-19 Pandemic.

The present study aimed to investigate family caregivers’ concerns about conducting ACP for home-dwelling individuals with dementia in Japan, which is experiencing both a super-aging society and the COVID-19 pandemic. We hypothesised that family caregivers whose loved ones with dementia had themselves initiated ACP would show a lower level of concern than those whose loved ones had not initiated ACP. Additionally, we sought to identify the variables, among caregivers and/or their loved ones, that predict high levels of caregiver concern regarding ACP. Our findings in Japan, where many older adults with dementia and family caregivers live in their communities under prolonged health crises, will provide implications for addressing the increasing needs for ACP initiation in other countries that also face an increasing number of people with dementia [14] and escalating needs for palliative care [15] under long-term restrictions, such as the COVID-19 pandemic.

## Methods

### Study design

A prospective cross-sectional design was adopted in this study using a web-based survey.

### Setting

Data were collected through an online survey conducted by an Internet survey company (Macromill Inc.) that manages a global online research system. On 25 June, 2021, a self-administered questionnaire was distributed (by sending e-mails and posting notifications on the survey company’s website) to individuals aged 40 years or older who had been randomly sampled from the company’s member pool. These individuals, provided they satisfied the eligibility criteria (see the ‘participants’ section below), were asked to complete the questionnaire by 27 June 2021.

Instructions for completing the questionnaire and information regarding the study were provided on the questionnaire website in advance of the presentation of the questionnaire items. This assured the participants that their personal information would be protected and that their data would be anonymised. Individuals who continued to the page with the questionnaire items were considered to have given consent to participate in this study. Previous research found that obtaining informed consent online is not substantially different from obtaining face-to-face consent [16, 17]. Any identifying information (participants’ names and other identifiers that could lead to the identification of a participant) was removed when we received the data from the Internet survey company, and no images/videos were obtained from the participants.

The web-based survey was adopted because face-to-face contacts with family caregivers of people with dementia had to be avoided during the pandemic. Furthermore, distribution of paper questionnaire may require obtaining the physical address of each member via some dementia-related organizations, which would challenge the protection of participants’ privacy. Online surveys with family caregivers of persons with dementia have been used previously in Japan [18] and other countries [19]. The study protocol was approved by the appropriate ethics review board and was conducted in accordance with the Helsinki Declaration of 1975 (as revised in 2013).

### Study size

We used G*Power 3.1.9.7 software to determine the necessary sample size for conducting an analysis of variance (ANOVA) for family caregiver concerns [20, 21]. Recent reports on the prevalence of ACP among people with dementia show substantial variation, from 11.8% in Belgium [22] to 48.0% in Australia [23]. In Japan, a national survey of end-of-life care conducted in 2017 reported that 22.5% of the respondents were aware of ACP [24]. Therefore, in this study, we assumed that the prevalence of ACP initiation among our sample was approximately 22.5%. Assuming a significance level of 0.05, a power of 95%, and a medium effect size (Cohen’s d = 0.5), and using a two-tailed test, the desired sample size was determined to be 350.

### Participants

The web-based survey was administered only to residents of Japan. The following inclusion criteria were set for participants: (i) aged 40 years or older, (ii) currently a primary non-professional caregiver for a person with dementia, and (iii) having no conflicts of interest through affiliation with advertising or marketing research entities. We excluded caregivers under the age of 40 years because such individuals comprise only 2% of all caregivers in Japan, making it difficult to appropriately determine the characteristics of such young caregivers [25]. Based on these criteria, the Internet survey company randomly recruited members from their potential pool of participants by sending e-mails and posting notifications on their website. It was estimated that the pool comprised 1,913 persons, who had previously declared that in their household, they had a family member with a dementia diagnosis, who regularly visited to healthcare organizations for treatment.

In the screening of potential participants, they were asked whether their loved one had received a diagnosis of Alzheimer’s disease, or vascular, Lewy body, or other types of dementias. If the loved one has had any dementia diagnosis, the family caregiver was also asked whether the loved one had regular visits to healthcare institutions for treatment. Therefore, in this survey, dementia was defined as having any diagnosis and receiving regular outpatient care for dementia.

Eligible individuals could access the self-report questionnaire after reading the terms and conditions of the online survey. As the Internet survey company ceased recruitment once the target number of respondents was reached, the response rate could not be determined. Individuals who completed the questionnaire received approximately 40 “Macromill points” as a reward for participation, which could be cashed in and used to shop online (one point was equivalent to one Japanese yen). This was in accordance with the ethics approval.

### Measurements

All variables were measured using an online self-report questionnaire that was developed by the authors. A total of 68 questions was used for the survey. The recruited participants were instructed to log into the survey company’s portal to complete the questionnaire.

Family concerns regarding having ACP-related conversations were assessed using six items developed by the present research team (Table 1). Respondents were asked to rate each item using a five-point Likert scale, ranging from ‘totally disagree’ to ‘totally agree’. The six statements were developed based on an analysis of literature regarding barriers to initiating ACP for people with dementia [9–12]. Respondents’ total scores for all six items were used for multivariate analysis. Higher total scores indicated greater concern regarding ACP conversations. The Cronbach’s alpha coefficient for this scale was 0.69.

**Table 1:** Family caregivers’ concerns about advance care planning (ACP) by ACP initiation and professional involvement

Mean (SD), range: 1–5	Professionals	Relatives only	Not initiated	F (2)	*P*-value
	*N* = 88	*N* = 67	*N* = 224		
I have no idea what or how to think about having a conversation with my family member with dementia regarding ACP	3.3 (1.1)	3.3 (1.1)^a^	3.6 (1.0)^a^	4.52	.012
I do not want to think about my family member’s end of life process	3.0 (1.2)	2.9 (1.2)	3.0 (1.2)	0.57	.564
I feel that I am not allowed to initiate a conversation about ACP with my family physician or other professionals who care for my family member	2.3 (1.0)^a^	2.5 (1.0)^b^	3.0 (1.0)^a,b^	13.43	< .001
I am concerned that initiating a conversation regarding ACP will cause emotional pain for my family member	2.9 (1.0)	3.1 (1.2)	3.1 (1.0)	0.97	.380
I do not exactly know what my family member’s values and goals are	2.9 (1.1)	3.2 (1.1)	3.2 (1.0)	2.33	.098
If I had dementia, I would like to initiate the conversation regarding my ACP^†^	4.2 (1.0)^a^	3.9 (1.1)^b^	3.5 (1.1)^a,b^	13.71	< .001
Sum of scores	16.3 (4.3)^a^	17.1 (4.3)^b^	18.5 (3.8)^a,b^	10.27	< .001

^a,^^b^ Significant difference with *P* < .017, Bonferroni correction

^†^Scoring is reversed

*ACP* Advance care planning, *SD* standard deviation

The respondents were also asked whether their loved ones had initiated ACP. ACP was defined as ‘the loved one thinking about his/her own future and talking to his/her family and others about what is important to him/her’. This definition was created by the research team based on materials from dementia-related associations [26, 27] and suggestions from family caregivers and staff working at four dementia-related organisations in Japan. Family caregivers who reported that their loved ones had initiated any form of ACP were asked to report the timing of the initiation (e.g. at diagnosis, hospital admission), the types of care professionals involved in the conversation (if any), and the topics discussed. The response categories for the item concerning the timing of the initiation were developed by the present research group based on existing recommendations for ACP concerning people with dementia [28, 29]. The types of professionals were defined based on consideration of dementia care pathways in Japan [30]. Seven response options were provided for the topics discussed; these included ‘important roles in the community and values’ and ‘implementation of tube-feeding when the person can no longer safely take food or fluid orally’. These response options were developed by the research group based on consideration of intervention programmes for encouraging ACP among community residents [31, 32]. As half of the respondents who reported ACP initiation stated that no care professionals were involved in the conversation, three groups were created: ‘never initiated ACP’ (N = 224), ‘no professionals other than relatives involved in ACP initiation’ (N = 67), and ‘care professionals were involved in the ACP-initiation conversation’ (N = 88).

We measured the characteristics of the respondents’ loved ones, including age, sex, type of dementia, time since clinical diagnosis, level of cognitive impairment, ADL ability, presence/absence of cancer, and participation in peer support groups. The time since diagnosis was categorised into ‘within 25 months’, ‘26–92 months’, or ‘93 months or longer’, respectively, based on the first and third quartiles of the responses. Level of cognitive impairment was evaluated using the Japanese version of the Cognitive Performance Scale (CPS), which was developed through the InterRAI Assessment System [33]. The CPS is a validated measure that uses five variables to classify older adults into categories in terms of cognition; the classifications range from ‘intact’ (a score of 0) to ‘very severely impaired’ (a score of 6) [33]. The Japanese version of the CPS has demonstrated fair reliability (weighted kappa = 0.77) and validity (Spearman’s correlations with the external scales = 0.8) [34]. ADL ability was measured using the Japanese version of the Activities of Daily Living Self-Performance Hierarchy Scale (ADL-H), again developed through the interRAI Assessment System [35]. The ADL-H is a 10-item scale that measures people’s ability to independently perform basic activities related to self-care and mobility; total scores range from 0 to 6, with higher scores indicating greater physical dependency. The Japanese version of the ADL-H has demonstrated good validity (Spearman’s correlations = 0.6–0.7 with external scales) [36]. In this study, participation in peer support groups was defined as participation in any of the following activities or societies: senior clubs, dementia cafés, Alzheimer’s Association Japan, and meeting centres for people with dementia.

We also assessed the characteristics of the family caregivers, including age, sex, educational attainment, relationship with the loved one, and psychological well-being. As the majority (74.9%) of the family caregivers were children of their loved ones, in the analysis the relationship with the loved one was categorised into ‘children’ and ‘other relatives’, respectively. The family caregivers rated their psychological well-being using the WHO-5 Well-Being Index, which contains five items that assess subjective psychological well-being [37]. Respondents were asked to rate how often each of the five statements applied to them during the past 14 days. Each of the five items is scored from 5 (‘all of the time’) to 0 (‘none of the time’). The total score ranges from 0 (‘worst thinkable well-being’) to 25 (‘best thinkable well-being’). The Japanese version of the WHO-5 has shown good reliability (Cronbach’s alpha = 0.87) and validity (Spearman’s correlation with Geriatric Depression Scale = -0.53) [38].

### Statistical analysis

Family concerns were compared among the three ACP-initiation groups. An ANOVA, using Bonferroni correction, was performed across the groups.

Multiple linear regression analysis was conducted using the total score for family-caregiver concerns as the dependent variable. All loved ones’ and family caregivers’ characteristics were included as covariates. Subgroup analyses were conducted by performing regression analysis for each ACP-initiation group.

All participants provided complete information; there were no missing values in the data. Statistical significance was set at α = 0.05. All statistical analyses were conducted using STATA, version 17.0 (StataCorp LLC, College Station, TX, USA).

## Results

### Participant characteristics

A total of 412 family caregivers registered with the Internet survey company completed the survey. Of the 412 respondents, 33 were excluded because they reported that their loved ones had been admitted to long-term care facilities or hospitals by the time of the survey. Therefore, the remaining 379 family caregivers of home-dwelling individuals with dementia were included in the final sample.

At the time of enrolment, the mean age of the family caregivers was 58.2 years (standard deviation [SD] = 8.9); 52.8% were men, 44.1% had graduated from university or graduate school, and the majority (74.9%) were children of the loved ones. Most (97.4%) of the respondents lived with their loved ones. One-fifth of the loved ones were men (19.0%; Table 2). Of the 200 family caregivers who were men, 85.5% were co-living caregivers of women with dementia.

**Table 2:** Participants’ characteristics (*N* = 379)

Variable	N (%) or mean (SD)
Family caregivers	
Age (years; range: 40–83), mean (SD)	58.2 (8.9)
Sex, male, n (%)	200 (52.8)
Educational attainment, n (%)	
Junior high school or high school	138 (36.4)
Vocational school or college	74 (19.5)
University or graduate school	167 (44.1)
Relationship with the person with dementia, n (%)	
Child	284 (74.9)
Spouse	61 (16.1)
Spouse of child	29 (7.7)
Other relative	5 (1.3)
Psychological well-being (range: 0–25), mean (SD)	10.8 (6.1)
Person with dementia	
Living situation	
Living with respondent caregiver	369 (97.4)
Living with another caregiver	3 (0.8)
Living alone	7 (1.8)
Age (years; range: 41–99), mean (SD)	82.7 (8.7)
Sex, male, n (%)	72 (19.0)
Time since diagnosis (months), range 2–313, mean (SD)	65.3 (55.8)
Type of dementia, n (%)	
Alzheimer’s disease	254 (67.0)
Lewy body	55 (14.5)
Vascular	51 (13.5)
Frontotemporal	9 (2.4)
Mixed	13 (3.4)
Other, including unspecified	24 (6.3)
ADL ability (range: 0–6), mean (SD)	2.9 (2.0)
Cognitive impairment (range 0–6), mean (SD)	3.1 (1.2)
Having cancer, n (%)	19 (5.0)
Participating in peer support groups, n (%)	68 (17.9)
Senior club	27 (7.1)
Alzheimer’s Association Japan	23 (6.1)
Dementia café	18 (4.7)
Meeting centre	7 (1.8)

*ADL* Activities of daily living, *SD* standard deviation

Activities of daily living were evaluated using the Japanese version of the Activities of Daily Living Self-Performance Hierarchy Scale

Cognitive impairment was evaluated using the Japanese version of the Cognitive Performance Scale

Psychological well-being was evaluated using the Japanese version of the World Health Organisation Five Well-Being Index (WHO-5)

### ACP initiation

One hundred and fifty-five (40.9%) of the respondents reported that their loved ones had initiated ACP. Of these, approximately half (49.7%) reported that the initiation was triggered by a dementia diagnosis. Two-fifths (43.2%) reported that no one other than relatives were involved in the ACP conversations. Regarding the cases in which health-care professionals were involved in such conversations, the most commonly reported type of professional was care managers of in-home care services (34.2%); the doctor who provided the dementia diagnosis was present in only 16.8% of the cases (Table 3).

**Table 3:** Timing of advance care planning (ACP) initiation and types of care professionals who were involved in the conversation

Of the 155 loved ones who initiated ACP	N (%)
Timing	
Upon receiving a clinical diagnosis of dementia	77 (49.7)
When being newly accredited for a long-term care insurance benefit	31 (20.0)
Began ACP before dementia diagnosis	18 (11.6)
Upon experiencing increased difficulty managing own property or daily life	13 (8.4)
Upon admittance to an acute hospital for treatment of a physical illness	7 (4.5)
Upon worsening physical health of relatives	6 (3.9)
Upon learning about ACP from peers or family caregivers	2 (1.3)
Upon learning of ACP through the media	1 (0.6)
Individuals involved in the ACP conversation	
No one other than relatives were involved	67 (43.2)
Care professionals (agencies)	88 (56.8)
Care manager of an in-home care service	53 (34.2)
Staff of a day-care centre	43 (27.7)
Care manager of a residential care service	39 (25.2)
Community General Support Centre^a^	27 (17.4)
The doctor who provided the clinical diagnosis of dementia	26 (16.8)
Peer with dementia	13 (8.4)
Staff of a dementia café or other meeting centres	11 (7.1)
Initial-phase Intensive Support Team for Dementia^b^	8 (5.2)
Doctor other than the one who provided the dementia diagnosis	3 (1.9)

^a^The Community General Support Centre provides comprehensive support for older community residents

^b^The Initial-phase Intensive Support team for Dementia conducts home visits and assessments, and provides information and advice to persons with early signs of dementia

The most frequently discussed ACP-related topic was the point at which the loved ones would need to enter residential care (67.7%). Other topics included important roles in the community and values (41.9%), the loved one’s habits and preferences (38.7%), social activities the loved one would like to continue (29.0%), social relationships the loved one would like to maintain (25.2%), tube feeding (25.2%), and application of cardiopulmonary resuscitation or transfer to an emergency department when breathing or heart stops (21.9%; Table 4).

**Table 4:** Topics discussed in advance care planning (ACP)

Of the 155 who initiated ACP	*N* (%)
The point at which the person would accept the need to enter residential care	105 (67.7)
Important roles in the community and values	65 (41.9)
The person’s habits and preferences	60 (38.7)
Social activities the person would like to continue	45 (29.0)
Social relationships the person would like to maintain	39 (25.2)
Implementation of tube feeding when the person can no longer safely take food or fluid orally	39 (25.2)
Application of cardiopulmonary resuscitation or transfer to an emergency department when breathing or heart stops	34 (21.9)

### Family caregivers’ concerns

The total caregiver sample had a mean score of 17.7 (SD = 4.1; range 6–30) for concerns. When compared with the 67 persons who reported no professional involvement in ACP conversations and the 224 persons who had not initiated ACP, the 88 persons who reported that care professionals were involved in the ACP conversations showed significantly lower levels of total concerns, concerns regarding talking with professionals, and concerns regarding commencing ACP for their own futures if they were to develop dementia (Table 1).

Multiple linear regression analysis showed that both longer time since dementia diagnosis and lower psychological well-being had significant associations with higher caregiver concerns (Table 5). In subgroup analyses of the three ACP-initiation groups, a significant association was found between caregiver concerns and psychological well-being among individuals from the ‘never initiated ACP’ group (coefficient =  − 0.23, 95% confidence interval [CI] =  − 0.32, − 0.15) and those from the ‘no professionals other than relatives involved in ACP initiation’ (coefficient =  − 0.31, 95%CI =  − 0.52, − 0.10). This association was not significant in the ‘care professionals were involved in the ACP-initiation conversation’ group (coefficient = 0.07, 95%CI =  − 0.11, 0.25). No other significant differences were found.

**Table 5:** Multiple linear regression analysis of family caregivers’ concerns regarding advance care planning (ACP) with persons with dementia

Variable	Coefficient	95%CI		*P*-value
		Lower	Upper	
Characteristics of the loved one				
Age (years)	− 0.02	− 0.07	0.04	.548
Sex, male	1.05	− 0.04	2.14	.060
Alzheimer’s disease	0.06	− 0.81	0.94	.886
Dependence for ADL (range: 0–6)	− 0.13	− 0.37	0.11	.276
Cognitive impairment (range: 0–6)	− 0.26	− 0.64	0.12	.180
Time since diagnosis (reference: < 25 months)				
26–92 months	0.29	− 0.70	1.28	.560
≥ 93 months	1.27	0.07	2.47	.038
Having cancer	0.31	− 1.58	2.21	.745
Participating in peer support groups	− 0.43	− 1.53	0.67	.445
Characteristics of the caregiver				
Age (years)	− 0.05	− 0.10	0.01	.093
Sex, male	0.64	− 0.25	1.53	.157
Child of the loved one	0.07	− 1.10	1.25	.904
Educational attainment (reference: junior high school or high school)				
Vocational school or college	− 0.76	− 1.89	0.37	.185
University or graduate school	− 0.35	− 1.29	0.59	.465
Psychological well-being (range: 0–25)	− 0.18	− 0.25	-0.11	< .001

*CI* confidence interval

Adjusted R^2^ = 0.08

Activities of daily living were evaluated using the Japanese version of the Activities of Daily Living Self-Performance Hierarchy Scale

Cognitive impairment was evaluated using the Japanese version of the Cognitive Performance Scale

Psychological well-being was evaluated using the Japanese version of the World Health Organisation Five Well-Being Index (WHO-5)

## Discussion

Overall, family caregivers of home-dwelling persons with dementia showed several types of ACP-related concerns. Their level of concern was significantly lower when the loved ones initiated ACP themselves. Family caregivers with lower psychological well-being showed significantly higher levels of concern; this association was also confirmed in subgroup analyses, albeit not for the ‘care professionals were involved in the ACP-conversation’ group.

The observed association between ACP initiation and low family-caregiver concerns aligns with our hypothesis. These results imply that family caregivers’ concerns may be barriers to the initiation of ACP for people with dementia, an association that has been suggested by several previous studies [9–12]. In particular, in the present study family caregivers from the ‘never initiated ACP’ group were more likely to be concerned about what and how to think about ACP, to feel unable to start a conversation with health-care professionals, and to be unwilling to initiate ACP for themselves should they develop dementia in the future. These concerns could have shaped psychological barriers to conducting ACP conversations. The concerns were especially high among family caregivers who exhibited poor psychological wellbeing. Thus, our study quantified and indicated an association between family caregivers’ concerns and psychological well-being. This association may be partly explained by the fact that the conversation process for ACP requires imagining a situation in which the loved one loses decisional capacity; such an event can represent a challenge for family caregivers [10], and family caregivers with poor psychological well-being may have little resilience to challenging circumstances and a low ability to negotiate uncertainty. However, trusting and open relationships can help caregivers overcome these difficult emotions [6, 28, 29, 39]. Therefore, educational and clinical strategies are needed to encourage professionals to address the psychological needs of family caregivers. In particular, asking open-ended questions can represent a good starting point for discussions on ACP, and can help identify the major themes family caregivers and their loved ones may be ruminating on [40]. Because a longer time since diagnosis was associated with higher family caregiver concerns regarding conducting ACP, such interventions should include family caregivers of persons with dementia who experienced a longer illness course as well as people who are newly diagnosed. Family caregivers’ experience with dementia may need to be further explored as hindering ACP conversations, because the individual’s care needs, including physical dependence and cognitive impairment, were not associated with the level of family caregiver concerns.

Our findings also showed that 41% of the participants’ loved ones had initiated ACP; this was a higher percentage than that included in our hypothesis (22.5%), which was based on data from a 2017 national report [24]. In 2018, the Ministry of Health, Labour and Welfare in Japan announced November 30^th^ as a national day of ACP (Jinsei-Kaigi), and this national campaign may have increased ACP awareness among community-dwelling older adults [41]. The uncertainty and instability in health care brought by the COVID-19 Pandemic may have also increased family caregivers’, as well as their loved ones’, awareness of the need to plan for the future.

Of the 155 loved ones who initiated ACP, only 57% did so in the presence of a professional caregiver who was not a relative of the loved one. Care managers of in-home care services (34%) who managed in-home care and coordinated with each in-home care service provider, and staff of day-care centres (28%) which provided personal care and day activities were the types of professionals (agencies) most commonly reported as being present during such conversations. Only 17% of the participants had conversations with the doctors who had provided the initial diagnosis of dementia. Regarding the topics discussed during such conversations, residential care occurred more frequently than end-of-life health care interventions. This indicates that there may be a discrepancy between existing ACP recommendations, which focus on recording patients’ wishes regarding the treatment process during the COVID-19 pandemic [42], and the topics persons with dementia and their family caregivers are actually worried about. This gap may have led to some family caregivers’ feeling that they are not allowed to start ACP conversations with doctors and other health-care professionals. Educational strategies and ACP tools may require psychosocial content that supports communication between persons with dementia, their family caregivers, and professionals [43, 44].

### Strengths and limitations

The strength of this study concerns its sampling of home-dwelling persons and the family-caregiver-centred outcome measures regarding ACP for people with dementia. However, our study also has some limitations. As it featured a cross-sectional design, causality between family-caregiver concerns and ACP initiation could not be determined. The relatively low concerns among the family caregivers whose loved ones had initiated ACP could have meant that the ACP conversations may have resulted in reduced family caregivers’ concerns about conducting ACP. Our data were based only on the responses of family caregivers, which may have biased the results. Since the respondents were recruited through the Internet with a small reward, the selection bias may limit the generalizability to the whole population. Although sex composition and mean age in our participants were similar with those reported in previous studies using online surveys in Japan [18, 45], our participants appeared to include more men and younger individuals than family caregivers of patients who used memory clinics in Japan [46]. Family caregivers show a low to moderate agreement with persons with dementia regarding care preferences (e.g. patients may feel, by discussing a certain topic, that they are discussing their future plans, while the caregivers may not recognise this topic as being associated with ACP) [47]. Hence, it is possible that the participants also show a low agreement with their loved ones regarding which conversations were about ACP or not. The ACP initiation, thus, could have been underreported by family caregivers. Furthermore, having dementia was assessed based on the family caregivers’ reports, which could not be confirmed by healthcare records. The ambiguity of dementia diagnosis might have led to underestimation of family caregivers’ concerns about conducting ACP.

## Conclusions

The COVID-19 Pandemic and the related restrictions have created an urgent need to initiate ACP for persons with dementia. However, some family caregivers have concerns regarding conducting ACP. The association between higher level of concerns and lower percentage of ACP initiation implies that family caregivers’ concerns could constitute barriers to initiating ACP conversations with their loved ones. Measuring the level of family caregivers’ concerns may inform their needs for psychosocial interventions to healthcare professionals in promoting ACP initiation for persons with dementia. In particular, caregivers with poor psychological wellbeing show greater concerns. This study is an important step toward improving post-diagnostic support for community-dwelling persons with dementia and their family caregivers, and underlines the need for addressing psychological barriers to initiating ACP. Educational and clinical strategies should be revised to encourage care professionals to address the psychological needs of family caregivers.

## Data Availability

The data that support the findings of this study are available upon request from the corresponding author. The data are not publicly available because of privacy and ethical restrictions.

## Abbreviations

ACP: Advance care planning
COVID-19: Coronavirus Disease 2019
